# Comparative Analysis of Gender-Specific Patterns of Traditional Coronary Artery Disease Risk Factors and QRISK3 Scores in First-Degree Relatives of Coronary Artery Disease Patients

**DOI:** 10.34172/jrhs.9005

**Published:** 2025-06-10

**Authors:** Meena Parmar, Pooja Vyas, Krutika Patel, Atisha Rana, Vijay Kalsariya, Mayuri Zadafiya

**Affiliations:** ^1^U.N. Mehta Institute of Cardiology and Research Centre, Civil Hospital Campus, Asarwa, Ahmedabad-380016, Gujarat, India; ^2^Department of Cardiology, U.N. Mehta Institute of Cardiology and Research Centre, Civil Hospital Campus, Asarwa, Ahmedabad-380016, Gujarat, India; ^3^Department of Research, U.N. Mehta Institute of Cardiology and Research Centre, Civil Hospital Campus, Asarwa, Ahmedabad-380016, Gujarat, India

**Keywords:** Coronary artery disease, Heart disease risk factors, Risk score

## Abstract

**Background:** Coronary artery disease (CAD) is a leading cause of death globally, with genetic and lifestyle factors contributing to its development. The first-degree relatives of CAD patients are at increased risk due to shared genetics and environments. This study aimed to perform a comparative analysis of gender-specific patterns of traditional CAD risk factors and QRISK3 scores in the first-degree relatives of CAD patients.

**Study Design:** A cross-sectional study.

**Methods:** This study enrolled 4,485 participants of first-degree relatives of patients who had been admitted to the hospital for CAD. Gender-specific comparisons were conducted in the first-degree relatives of CAD to assess traditional risk factors and QRISK3 scores.

**Results:** The mean age of patients was 41.8 years, with males comprising 66% of participants in our study. Males exhibited more traditional risk factors, including higher systolic and diastolic blood pressure, dyslipidaemia, smoking, alcohol, junk food consumption, and oral tobacco use. Females had a higher prevalence of obesity, inadequate sleep, depression, and migraines. Males had a significantly higher 10-year CAD risk according to QRISK3 scores, reflecting an increased healthy heart age of 4.20±1.32 years compared to their chronological age (*P*=0.0004).

**Conclusion:** The prevalence of coronary risk factors was twice as high in the first-degree male relatives of individuals with known CAD compared to females. Distinct gender-based differences were found in risk factors and QRISK3 scores, highlighting the significance of specific approaches in evaluating and managing the risk of CAD within this high-risk group.

## Background

 Globally, cardiovascular disease (CVD) represents a major public health issue as it considerably increases morbidity and mortality. The complexity of inherent risk factors for coronary artery disease (CAD) can vary significantly based on gender and familial predisposition. Asian Indians have a 20–50% higher relative risk of CAD-associated mortality than other ethnic backgrounds.^1^ The Framingham Heart Study and its subsequent offspring cohorts have shown that having a first-degree relative with CAD significantly increases an individual’s likelihood of developing the disease.^2^ The first-degree relatives of premature CAD patients have a heightened cardiovascular risk burden, especially with aging, as shown by risk assessment tools, underscoring that family history is a substantial risk factor. Studies have consistently demonstrated that a family history of premature CAD is a significant independent risk factor.^3-5^

 The prevalence of traditional CAD risk factors among the first-degree relatives of CAD patients in India reveals significant gender-specific differences.^3,6,7^ Common risk factors include high blood pressure, cholesterol levels, smoking, diabetes, obesity, and physical inactivity, but their prevalence and impact vary by gender. While males and females share similar rates of hypertension (HTN) and diabetes, studies have noticed differences in various risk factors in North Indian populations. For instance, North Indian females, especially at older ages, exhibit a higher incidence of diabetes and HTN, along with lower levels of dyslipidaemia and body mass index (BMI), compared to males.^6,7^ Dyslipidaemia is more prevalent in young males compared to females, indicating a gender disparity in lipid profiles.^8^ Conversely, obesity rates are higher among females, a critical CAD risk factor, as per previous research.^9^ A small sample size and age matching between the two groups would substantiate the difference in the risk factor profile. Previous research reported that healthier siblings still exhibit a concerning prevalence of cardiovascular risk factors, emphasizing the familial component of CAD risk.^7^

 The QRISK3 score, an advanced prediction algorithm, integrates these traditional risk factors with additional variables, such as kidney function, atrial fibrillation, certain psychological conditions, and certain inflammatory health conditions, to estimate the 10-year risk of developing CAD.^10,11^

 This comparative analysis highlights the importance of evaluating traditional risk factors and QRISK3 scores, specifically within a population subset—first-degree relatives (parents, siblings, or children) of CAD patients. This population is of particular importance because genetic factors significantly contribute to the risk of developing CAD. By examining both male and female first-degree relatives separately, the study aims to discover any gender-specific patterns in traditional CAD risk factors and QRISK3 scores. By exploring conventional risk factors alongside QRISK3 scores, which incorporate additional variables for a more comprehensive risk assessment, the study seeks to contribute to a more nuanced understanding of the potential hereditary and gender-related variations in CAD risk.

## Methods

###  Sampling and study population

 This cross-sectional study was conducted at a tertiary cardiac care hospital in Ahmedabad. The study population was the first-degree relatives of patients who had been admitted to the hospital for CAD. The study protocol was approved by the institutional ethics committee. Written informed consent was obtained from all participants.

###  Study protocol

 The study was performed between October 2022 and October 2023. Participants who were more than 18 years old and in good general health or with stable chronic conditions were included in the study. On the other hand, all the first-degree relatives with known clinical history and evidence of acute coronary syndrome or chronic stable CAD diagnosed on stress coronary testing or coronary angiography were excluded from the study.

 In this study, 4,485 first-degree relatives were included, and a standardized pre-designed form was used to collect required data, including demographic information, age, gender, socioeconomic condition, height, weight, and blood pressure. BMI was calculated using weight in kg/height in m^2^. According to the World Health Organization, a BMI greater than or equal to 30 kg/m^2^ is considered obesity.^12^ The medical officer utilized a sphygmomanometer to take blood pressure on the right upper arm while seated. Three 1-minute-interval readings were taken, and the average of the previous two values was considered for final evaluation. The participant’s medical and family history was noted, including information about HTN, type 2 diabetes mellitus, hypothyroidism, and chronic renal disease. The habits of eating food, tobacco chewing, alcohol intake, and physical activities were also recorded, according to standard questionnaires.

 For laboratory findings, blood samples were drawn to test the complete blood count, serum creatinine, fasting blood sugar, and fasting lipid profile using the International Federation of Clinical Chemistry-approved enzymatic methods on an auto-analyzer by a commercially available kit (ARCHITECH PLUS ci4100, Germany).

###  Validity assessment

 Data collection questionnaires are used to assess an individual’s or population’s quality of life, which includes a variety of well-being indicators. Face validity was evaluated by independent specialists (three cardiologists and four public health experts). Participants were asked to ensure that the evaluation tool covered all relevant domains.

 Dyslipidaemia was defined as total cholesterol > 200 mg/dL, low-density lipoprotein cholesterol > 130 mg/dL, high-density lipoprotein cholesterol < 40 mg/dL in males and < 50 mg/dL in females, and triglycerides > 150 mg/dL.^13^ Socio-economic conditions in Gujarat were evaluated using the Below Poverty Line and Above Poverty Line criteria, as defined by the Government of Gujarat under the National Food Security Act-2013.^14^ Regular exercise was defined as at least 30 minutes per day moderate-intensity exercise for at least 5 days a week.^15^ Yoga was defined as at least 15–20 minutes of yoga for 5 days a week. Eating junk food was defined as 3 times a week or more consumption of junk food.

###  Risk score calculation

 Apart from the above information collected, the 10-year risk of having a major CV event (cardiovascular death, myocardial infarction, or stroke) was calculated for each patient using the QRISK3 risk score calculator (https://www.qrisk.org/).^16^ The risk increases with a higher risk score. In addition, the risk is greater when there are more risk factors. The QRISK3 risk score was estimated using participants’ self-reported risk factors with standard questionnaires filled out by a medical officer. Participants with less than 25 years (n = 371) were excluded from the QRISK3 score calculation. The QRISK3 risk score was computed for 4,114 out of 4,485 subjects. The QRISK score is used to predict the 10-year risk of CVD. The risk level is categorized into low-risk (less than 10%), intermediate-risk (10–20%), and high-risk (over 20%) groups.

###  Statistical analysis

 Statistical analyses were performed using SPSS, version 26.0 (Chicago, USA). Continuous and categorical variables were expressed as means ± standard deviations (SD), as well as numbers and percentages, respectively. Moreover, the chi-square test and Student’s t-test were utilized for categorical and continuous variables, respectively. Additionally, a logistic regression model was employed to evaluate the association between reported risk factors of CAD and the 10-year risk of CVD occurrence. A *P* < 0.05 was considered statistically significant.

## Results

 In the study, 4,485 participants were enrolled, including 2,958 (66%) male and 1,527 (34%) female participants. The mean age of the participants was 41.8 years. Demographic details of all participants are mentioned in Table 1. The results demonstrated an association between gender-specific risk variables. Males were more likely to have traditional risk factors than females. The mean ( ± SD) age of males and females was 41.08 ( ± 12.17) and 43.14 ( ± 12.39), respectively (*P* < 0.001). Systolic (131.08 ± 16.82 vs. 127.65 ± 19.48, *P* < 0.001) and diastolic (85.90 ± 11.52 vs. 83.69 ± 11.68, *P* < 0.001) blood pressure were significantly higher in males than in females. The presence of 3 or more cardiac risk factors significantly increased in males compared to females (*P* < 0.004).

**Table 1:** Demographic details and gender-specific comparison

**Continuous Variables**	**Male (n=2958)**	**Female (n=1527)**	**Total (N=4485)**	
	**Mean (SD)**	**Mean (SD)**	**Mean (SD)**	***P *** **value**
Age (years)	41.08 (12.17)	43.14 (12.39)	41.78 (12.29)	0.001
Systolic blood pressure (mm Hg)	131.08 (16.82)	127.65 (19.48)	129.91 (17.85)	0.001
Diastolic blood pressure (mm Hg)	85.9 (11.52)	83.69 (11.68)	85.15 (11.62)	0.001
BMI (kg/m^2^)	24.49 (4.22)	26.44 (26.44)	25.15 (4.65)	0.001
**Categorical variables**	**Number (%)**	**Number (%)**	**Number (%)**	***P *** **value**
Type 2 diabetes mellitus	729 (24.6)	369 (24.2)	1098 (24.5)	0.750
Hypertension	777 (26.3)	414 (27.1)	1191 (26.6)	0.570
Triglyceride ( > 150 mg/dL)	920 (31.1)	358 (23.4)	1278 (28.5)	0.001
Low-density lipoprotein ( ≥ 140 mg/dL)	806 (27.2)	236 (15.5)	1042 (23.2)	0.001
High-density lipoprotein ( < 40 mg/dL)	1674 (56.6)	509 (33.3)	2183 (48.7)	0.001
Dyslipidaemia	2183 (73.8)	805 (52.7)	2988 (66.6)	0.001
Obesity (BMI > 30 kg/m^2^)	1329 (44.9)	920 (60.2)	2249 (50.1)	0.001
Rheumatoid arthritis	0	10 (0.7)	10 (0.2)	0.001
Depression/mental illness	70 (2.4)	150 (9.8)	220 (5)	0.001
Migraine	38 (1.3)	76 (5)	114 (2.5)	0.001
2 or more risk factors	1669 (56.4)	780 (51.1)	2449 (54.6)	0.007
3 or more risk factors	918 (31)	409 (26.8)	1327 (29.6)	0.004
History of addictions				
Alcohol	196 (6.6)	1 (0.1)	164 (3.7)	0.001
Smoking	1873 (63.3)	3 (0.2)	312 (7)	0.001
Oral tobacco consumption		17 (1.1)	893 (19.9)	0.001
Lifestyle factors	1079 (36.5)			
Inadequate sleep ( < 7 hours/day)	80 (2.7)	162 (10.6)	358 (8)	0.001
Junk food consumption		853 (55.9)	2726 (60.8)	0.001
Physical activity	371 (12.5)			
Exercise ( ≥ 30 minutes/day)	2587 (87.5)	419 (27.4)	1498 (33.4)	0.001
Yoga (5 days a week)	196 (6.6)	45 (2.9)	125 (2.8)	0.710
Personality	1873 (63.3)			0.010
Type A		151 (9.9)	522 (11.6)	
Type B	1079 (36.5)	1376 (90.1)	3963 (88.4)	

*Note*. SD: Standard deviation; BMI: Body mass index.

 Overall, 48.7% of patients had low level of high-density lipoprotein (33.83 ± 4.09 mg/dL), and 28.5% of them suffered from hypertriglyceridemia (225.83 ± 103.45 mg/dL). In total, 66.6% of the patients had dyslipidaemia (73.8% males vs. 52.7% females, *P* < 0.001). Junk food consumption habits were also more prevalent in males (63.3% vs. 55.9%, *P* < 0.001). History of smoking, drinking alcohol, and oral tobacco consumption were also more noticeable in males (30.6% vs. 1.4%, *P*< 0.001). In our study, the type A personality was significantly more prevalent in males than in females (12.5% vs. 9.9%, *P* < 0.01). The prevalence of type 2 diabetes mellitus (24.6% vs. 24.2%, *P* < 0.75) and HTN (27.1% vs. 26.3%, *P* < 0.57) was almost similar in both genders. The prevalence of regular exercise was more considerable in males than in females (36.5% vs. 27.4%, *P* < 0.001), despite exhibiting other risky behaviours, such as smoking, oral tobacco and alcohol consumption, and habits of eating more outside. The practice of yoga was more prevalent in females, with no statistically significant difference (*P*< 0.71).

 Females had a significantly higher prevalence of obesity (60.2% vs. 44.9%, *P* < 0.001), inadequate sleep ( < 7 hours/day, 10.6% vs. 6.6%, *P* < 0.001), depression or mental illness (9.8% vs. 2.4%, *P* < 0.001), and migraines (5% vs. 1.3%, *P* < 0.001).

 Based on the analysis of the 10-year CAD risk through the QRISK3 calculator, participants aged less than 25 years (n = 371) were excluded from the calculations. The 10-year CAD risk (N = 4114) was analyzed based on age, gender, and the same ethnic population. Males had elevated risk factors of QRISK3 compared to females (*P* < 0.001, Table 2). This was also reflected in the difference between chronological and healthy heart age for females, which was less as compared to males (*P* < 0.0004). The healthy heart age of males was 4.2 ± 1.32 years more compared to their chronological age (*P* < 0.001). Gender differences in QRISK3 scores and healthy heart age had significant implications for cardiovascular risk assessment and prevention. Males generally have higher QRISK3 scores at younger ages, indicating higher short-term cardiovascular risk, while females’ risk tends to increase later in life.

**Table 2:** Assessment of QRISK3 With Gender Comparison

**QRISK3 Score**	**Males (n=2742)**	**Female (n=1372)**	**Total (N=4114)**	***P*** ** value**
**Categorical variables**	**Number (%)**	**Number (%)**	**Number (%)**	
Low risk ( < 10%)	2193 (80.0)	1199 (87.4)	3392 (82.5)	0.001
Moderate risk (10-20%)	365 (13.3)	131 (9.5)	496 (12.0)	0.001
High risk ( > 20%)	184 (6.7)	42 (3.1)	226 (5.5)	0.001
**Continuous variables**	**Mean (SD)**	**Mean (SD)**	**Mean (SD)**	***P *** **value**
10-year risk of coronary artery disease (%)	6.05 (8.26)	4.53 (5.60)	5.55 (7.52)	0.001
Healthy heart age (years)	46.75 (12.68)	49.59 (13.36)	47.68 (13.20)	0.001
Mean chronological age (years)	42.55 (11.36)	45.62 (10.49)	43.58 (11.20)	0.001
Difference of age (years)	4.20 (1.32)	3.97 (2.87)	4.10 (2.00)	0.001

*Note*. SD: Standard deviation.

**Figure 1:**
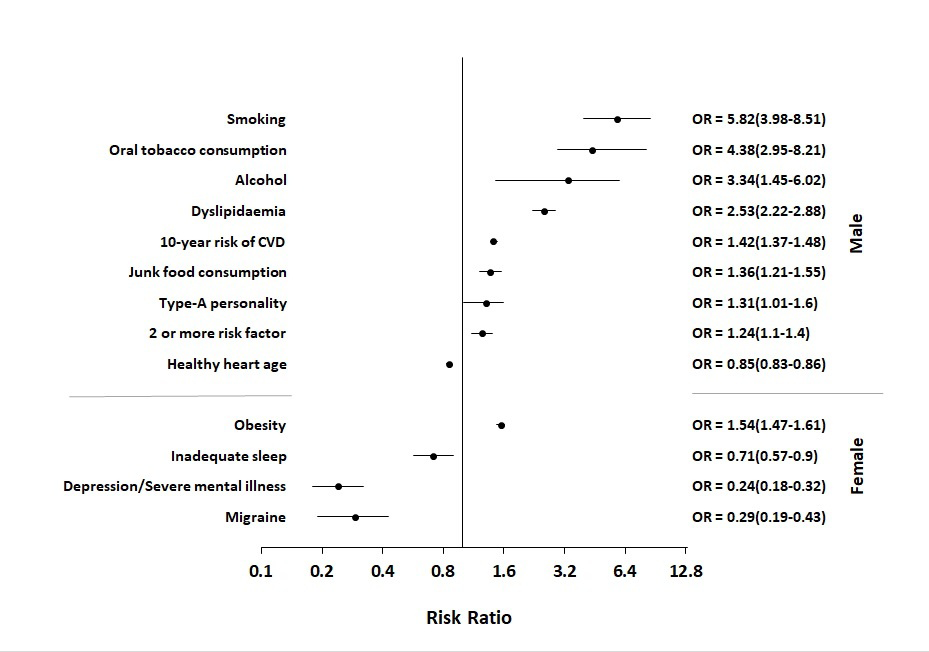
Forest plot for risk ratio. *Note*. CVD: Cardiovascular disease. This forest plot displays odds ratios with their associated 95% confidence levels, with a vertical line at no effect indicator. Markers to the left of this line represent reduced risk, while those to the right indicate increased risk

 The association between traditional CAD risk factors and the occurrence of CVD in first-degree relatives was evaluated using logistic regression models. According to our logistic regression models, smoking (odds ratio [OR]: 5.82, 95% confidence interval [CI]: 3.98–8.51, *P* < 0.001), oral tobacco consumption (OR: 4.38, 95% CI: 2.95–8.21, *P* < 0.001), alcohol (OR: 3.34, 95% CI: 1.45–6.02, *P* < 0.001), dyslipidaemia (OR: 2.53, 95% CI: 2.22–2.88, *P* < 0.001), junk food consumption (OR: 1.36, 95% CI: 1.21–1.55, *P* < 0.001), type-A personality (OR: 1.31, 95% CI: 1.01–1.6, *P* = 0.009), presence of two or more risk factors (OR: 1.24, 95% CI: 1.10–1.40, *P* = 0.0007), and 10-year risk of CAD (OR: 1.42, 95% CI: 1.37–1.48, *P*< 0.001) were significantly associated with the male gender. Our regression analysis revealed that the male gender, smoking, oral tobacco consumption, alcohol consumption, dyslipidaemia, type-A personality, and 10 years of CAD risk were significantly related to future CAD events. However, obesity (OR: 1.56, 95% CI: 1.49–1.64, *P*< 0.001) was found to be an independent risk factor for the female gender associated with future CAD events (Table 3 and Figure 1).

**Table 3:** Regression analysis of gender-specific risk factors

**Variables**	**Odds Ratio (95% CIs)**	***P*** ** value**
Male		
Smoking	5.82 (3.98–8.51)	0.001
Oral tobacco consumption	4.38 (2.95–8.21)	0.001
Alcohol	3.34 (1.45–6.02)	0.001
Dyslipidaemia	2.53 (2.22–2.88)	0.001
10-year risk of coronary artery disease	1.42 (1.37–1.48)	0.001
Junk food consumption	1.36 (1.21–1.55)	0.001
Type-A personality	1.31 (1.01–1.60)	0.009
2 or more risk factors	1.24 (1.10–1.40)	0.001
Healthy heart age	0.85 (0.83–0.86)	0.001
Female		
Obesity	1.54 (1.47–1.61)	0.001
Inadequate sleep	0.71 (0.57 –0.90)	0.004
Depression/severe mental illness	0.24 (0.18–0.32)	0.001
Migraine	0.29 (0.19–0.43)	0.001

*Note*. CI: Confidence interval.

## Discussion

 This study explored the gender-specific patterns in CAD risk factors and comparative results of QRISK3 scores between genders. Traditional CAD risk factors are common to both males and females, and there are differences in the prevalence, impact, and manifestation of these factors based on gender.^17-20^

 The Indian population faces a substantially increased risk of heart disease, attributed to a mix of genetic, cultural, lifestyle, and economic influences. People of South Asian descent are more likely to be genetically predisposed to conditions such as insulin resistance, central obesity, and type 2 diabetes. According to research, those with South Asian ancestry may be prone to developing heart disease at a younger age and with a lower BMI than those in Western populations.^19^ According to Sah et al, coronary risk factors were different, and the effects of all variables were population-specific in their ethnicity.^21^ In the Indian population, the risk profile of CVD was increased in males owing to the continuing development of the atherosclerotic process.^20,22-24^ Dyslipidaemia was significantly higher in males than in females (*P* < 0.001). India has a high rate of tobacco consumption, particularly through smoking and chewing tobacco. Chewing tobacco is linked to cardiovascular risk. Alcohol consumption is also rising in India and is associated with an increased risk of CVD.^20^

 The study by Aggarwal et al found that males had a higher risk of CVD since they had more frequent habits of smoking, tobacco consumption, and alcohol drinking,^17^ which is in line with our findings. Some studies reported that CAD risk factors were significantly higher in males than in females.^2,10,25^

 Blum and Blum found that the prevalence of CAD was significantly lower in females < 60 years than in older females. Moreover, the CAD prevalence rate was elevated, and the exceeded level of CAD risk was reported in males.^26^ Previous studies and meta-analyses demonstrated that females had a higher prevalence of modified and non-modified risk factors, such as HTN, obesity, depression, migraine, and sedentary lifestyle, than males due to the combination of biological, sociocultural, and economic factors. Obesity and mental health issues are closely linked.^27,28^ Depression and anxiety can lead to eating more, sedentary lifestyles, and further weight gain. Sedentary occupations, limited recreational exercise, and reliance on motorized transport contribute to obesity and metabolic syndrome.^23,27^ In our study, females also had a higher prevalence of obesity, depression, migraine, and sedentary lifestyle.^20,28-30^

 Dietary habits and physical activity are associated with a lower prevalence of atherosclerotic activity and metabolic syndrome. Many traditional Indian diets are heavy on refined carbs, saturated fats, and salt, often contributing to health concerns. Certain cultural practices, such as oily and fatty foods used in North India, can exacerbate cholesterol levels. Even though vegetarian diets are popular, they can be lacking in protective nutrients, such as omega-3s, if not carefully balanced. The sharp shift from traditional meals to Western-style fast foods in cities can also significantly increase cardiovascular risk.^6,7,21^

 Khatun et al concluded that consumption of junk foods was associated with a significantly higher risk of CADs.^31^ Our findings revealed that 60.8% of the population had habits of eating junk food. According to the 2018 Physical Activity Guidelines, 150–300 minutes of weekly moderate-intensity aerobic exercise or 75–150 minutes of weekly intense exercise led to a 40% reduction in the incidence of CVD and its associated risk factors.^32^ Furthermore, seventeen meta-analyses and one systemic review, including 594 129 individuals, provided evidence that physical activity reduces the progression of HTN.^27^ One more study conducted by Zenab et al reported a statistically significant inverse relationship between physical activity and cumulative CVD risk factors.^11^ Based on the results of our study, only 33.4% of the population were doing regular physical exercise.

 Depression can deteriorate cardiac function. According to our findings, 5% of our study population suffered from depression. The INTERHEART trial found that self-reported psychosocial variables were independently associated with the risk of an acute myocardial infarction.^33^ The quality of sleep can also affect cardiovascular risk. A meta-analysis of 74 studies involving 3 340 684 participants demonstrated that those who did not have adequate sleep (7–8 hours) were more likely to develop CVD.^34^ In our study, 10.6% of females reported inadequate sleep as compared to 6.6% of males.

 The 10-year CVD risk was evaluated using the QRISK3 score. According to the results of the current study, 82.5% of the population was at low risk for CVD over the next ten years. Out of the 4,114 individuals with a 10-year CVD risk of > 10%, 549 (20%) were male, and 173 (12.6%) were female. Based on the statistical analysis, a high ( ≥ 20%) 10-year QRISK3 risk score was shown to be statistically significant (*P* < 0.001) and was related to the male gender. Agrawal et al, Zenab et al, and Chandrawanshi et al reported equivalent conclusions in their studies. They found a correlation between the QRISK3 risk score and CVD risk factors.^10,11,35^ Our findings confirmed a significant difference of 4.97 ± 2.87 years between healthy heart age and chronological age of female participants as compared to 4.20 ± 1.32 years of difference in male participants. The evidence of gender-based distinctions in heart age necessitates tailored public health messages to address specific risk factors and their progression in males and females. It provides little evidence that lifestyle behaviour change can improve clinical outcomes. Males tend to have higher cardiovascular risk scores at younger ages, largely due to lifestyle factors.^8,9^ Females initially have lower scores, but risk rapidly increases after menopause. Current risk models often overlook females’ unique risk factors, leading to bias.

 This study had some limitations. This cross-sectional study was conducted at a single tertiary care hospital, which may limit the generalizability of the findings to other populations or settings. Self-reported data introduce potential biases related to recall, social desirability, and subjective interpretation of symptoms, affecting the accuracy of risk factor assessment. Improper averaging of male and female prevalence rates would underestimate the true prevalence.

 The study may not have fully accounted for confounding variables, such as dietary habits, physical activity levels, stress, medication use, and comorbid conditions (e.g., diabetes, HTN, or dyslipidaemia). Without adjusting for these confounders, the observed gender-specific differences in CAD risk factors and QRISK3 scores could be influenced by external factors rather than true biological or genetic predisposition.

**Highlights**

- The study focused on the QRISK3 scores and traditional coronary artery disease (CAD) risk factors of first-degree relatives of CAD patients.
- The first-degree male relatives of known CAD patients had 2-fold increased coronary risk factors.
- The study highlights the importance of developing gender-specific strategies for assessing and managing cardiovascular risks due to differences.

## Conclusion

 The first-degree male relatives of known CAD patients had a 2-fold increased prevalence of coronary risk factors compared to females. There were distinct gender-based differences in risk factors and QRISK3 scores, highlighting the significance of specific approaches in the evaluation and management of the risk of CAD within this high-risk group.

 These research findings suggest the need for targeted interventions that address gender-specific conditions with education and preventive measures tailored to each gender. It is crucial for future studies to confirm these findings. In addition, long-term research will allow a more thorough understanding of how gender affects CAD development and risk factor interactions. This results in a robust evidence base, enabling tailored interventions to improve patient outcomes.

## Acknowledgments

 We express our sincere gratitude to all individuals and organizations who contributed to this study. In addition, special thanks go to the medical officers and research department for their invaluable assistance.

## Competing Interests

 The authors declare that they have no conflict of interests.

## Ethical Approval

 The study has been approved by the institutional ethics committee (UNMICRC/CARDIO/2022/25).

## Funding

 The authors received no financial support for the research, authorship, and/or publication of this article.
